# Néel‐Vector‐Dependent Unconventional Spin‐Orbit Torque for Deterministic Field‐Free Switching in NiO (110)‐Based Trilayers

**DOI:** 10.1002/advs.76444

**Published:** 2026-07-09

**Authors:** Hyeong‐Joo Seo, Seok‐Jong Kim, Phuoc Cao Van, Hong‐Cuong Truong, Dong‐Hyeon Han, Geunwoo Kim, Younghun Jo, Jong Hoon Jung, Soojung Kim, Chun‐Yeol You, Jong‐Ryul Jeong, Kyung‐Jin Lee, Byong‐Guk Park

**Affiliations:** ^1^ Department of Materials Science and Engineering KAIST Daejeon South Korea; ^2^ Department of Physics KAIST Daejeon South Korea; ^3^ Department of Materials Science and Engineering Chungnam National University Daejeon South Korea; ^4^ Korea Basic Science Institute Daejeon South Korea; ^5^ Department of Physics Inha University Incheon South Korea; ^6^ Department of Physics and Chemistry Daegu Gyeongbuk Institute of Science and Technology (DGIST) Daegu South Korea

**Keywords:** antiferromagnet, field‐free, magnetization switching, spin pumping, spin‐orbit torque, unconventional SOT

## Abstract

Spin‐orbit torque (SOT) provides an efficient route for ultrafast and energy‐efficient magnetization control in spintronic devices. However, deterministic switching of perpendicular magnetization in conventional non‐magnet/ferromagnet bilayers is fundamentally limited by symmetry, typically necessitating an external magnetic field. In this work, we demonstrate unconventional spin currents and associated SOTs in antiferromagnet (AFM)‐based NiO(110)/Ta/CoFeB/MgO trilayers, enabling nearly complete field‐free switching of perpendicular magnetization. We find that the switching efficiency is maximized when the applied current is oriented transverse to the AFM easy axis, indicating that the generated spin currents are governed by the relative orientation between the Néel vector and the current direction. Second‐harmonic measurements reveal angle‐dependent spin currents with both in‐plane and out‐of‐plane spin polarizations, which is consistent with micromagnetic simulations incorporating the Néel‐vector dynamics in the AFM layer. Our findings suggest that AFM‐based trilayers offer a versatile platform for efficient field‐free switching and highlight the potential of Néel‐vector‐dependent spin currents for SOT‐based spintronic applications.

## Graphical abstract

Unconventional spin currents are generated in an antiferromagnetic NiO(110)/Ta/CoFeB trilayer, where the Néel vector governs spin transport. The resulting spin–orbit torques enable deterministic field‐free switching of perpendicular magnetization. The generated spin currents with both in‐plane and out‐of‐plane spin polarizations exhibit distinct angular dependences, highlighting the active role of antiferromagnetic spin dynamics.

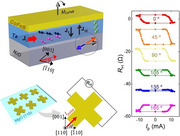

## 1 Introduction

Spin‐orbit torque (SOT) has garnered significant attention as a key mechanism for achieving ultrafast and energy‐efficient magnetization control in spintronic devices [1, 2, 3]. In particular, sub‐nanosecond switching of perpendicular magnetic tunnel junctions has been demonstrated using SOT [4, 5]. However, deterministic SOT switching in conventional non‐magnetic metal (NM)/ferromagnet (FM) bilayer structures remains fundamentally constrained by symmetry because spin currents generated via the spin Hall or Rashba‐Edelstein effects carry in‐plane spin polarization. While this constraint depends on the SOT geometry, it remains a key challenge for switching perpendicularly magnetized systems without an external magnetic field [6]. To overcome this limitation, various strategies have been proposed to achieve field‐free SOT switching. These include introducing effective magnetic fields via exchange coupling [7], exchange bias [8, 9], anisotropy gradient [10], or interlayer Dzyaloshinskii–Moriya interaction (DMI) [11], as well as breaking lateral symmetry either extrinsically through structural engineering [12, 13, 14, 15] or intrinsically by employing non‐centrosymmetric materials [16, 17].

Beyond NM layers, spin currents can also be generated within FM layers through both the conventional spin Hall effect and the magnetic spin Hall effect [18, 19]. The former produces spin currents with in‐plane spin polarization, similar to NM layers, whereas the latter enables the generation of spin currents with out‐of‐plane spin polarization when the charge current is applied parallel to the magnetization direction. FM layers can also generate spin currents via interfacial spin‐orbit filtering or spin‐orbit precession effects [18, 19], producing in‐plane or out‐of‐plane spin polarizations, respectively. These FM‐based spin currents can be exploited in magnetic trilayer structures composed of FM/NM/FM stacks [20, 21, 22, 23], where spin currents generated from the bottom in‐plane FM carry both in‐plane and out‐of‐plane spin polarizations, thereby enabling field‐free switching of the top perpendicular FM layer. Notably, the presence of an out‐of‐plane spin polarization not only enables field‐free SOT switching but also significantly reduces the switching current density [24].

In parallel, antiferromagnet (AFM) materials have emerged as an alternative and highly attractive spin current source. Despite having negligible net magnetization, AFMs can generate transverse spin currents through the antiferromagnetic spin Hall effect [25, 26, 27]. In this mechanism, the spin polarization of the generated spin current is governed by the orientation of the Néel vector relative to the applied current direction. When a charge current is applied transverse to the Néel vector, a spin current with out‐of‐plane polarization can be generated. Importantly, the utilization of AFMs as spin current sources offers intrinsic advantages, including robustness against external magnetic fields and minimal perturbation from neighboring magnetic elements.

Another approach to generating spin currents in AFMs is spin pumping driven by the Néel‐vector dynamics, which is the antiferromagnetic analogue to FM spin pumping [28]. AFM spin pumping has been demonstrated in various systems, in which AFM moments are excited by high‐frequency stimuli, such as optical pulses [29, 30], microwave radiation [31, 32, 33], or microwave currents [34]. In these cases, the spin polarization of the pumped spin current is determined by the oscillation axis of the Néel vector. Moreover, it has been theoretically proposed that Néel‐order dynamics and the associated AFM spin pumping can also be driven by a DC current via SOT [35, 36]. The realization of such SOT‐driven AFM spin pumping would eliminate the need for external high‐frequency excitation, thereby simplifying device structures and facilitating practical AFM‐based spintronic devices. Furthermore, when insulating AFMs are employed, parasitic current shunting within the AFM layer can be completely avoided, in contrast to the FM trilayers, providing an additional advantage for realizing energy‐efficient and scalable field‐free SOT devices.

In this work, we demonstrate unconventional spin currents and the associated SOTs in AFM‐based trilayers, where the AFM plays an active role as a spin current source. Using an AFM NiO(110)/Ta/CoFeB trilayer, we achieve deterministic field‐free SOT switching of perpendicular magnetization and observe a clear angular dependence of the switching efficiency, which is maximized when the applied current is oriented transverse to the AFM easy axis, consistent with anomalous Hall loop measurements. These results indicate that the generated spin currents are governed by the Néel‐vector orientation relative to the current direction. Furthermore, second‐harmonic measurements reveal the coexistence of in‐plane and out‐of‐plane spin polarizations with distinct angle dependences, which are in good agreement with micromagnetic simulations based on Néel‐vector dynamics in the AFM layer. Together, these findings suggest that AFM‐driven spin dynamics may contribute to the observed unconventional SOTs and establish AFM‐based trilayers as a versatile platform for efficient field‐free SOT switching in spintronic applications.

## 2 Results and Discussion

### 2.1 Field‐Free SOT Switching in a NiO‐Based Trilayer

**FIGURE 1:**
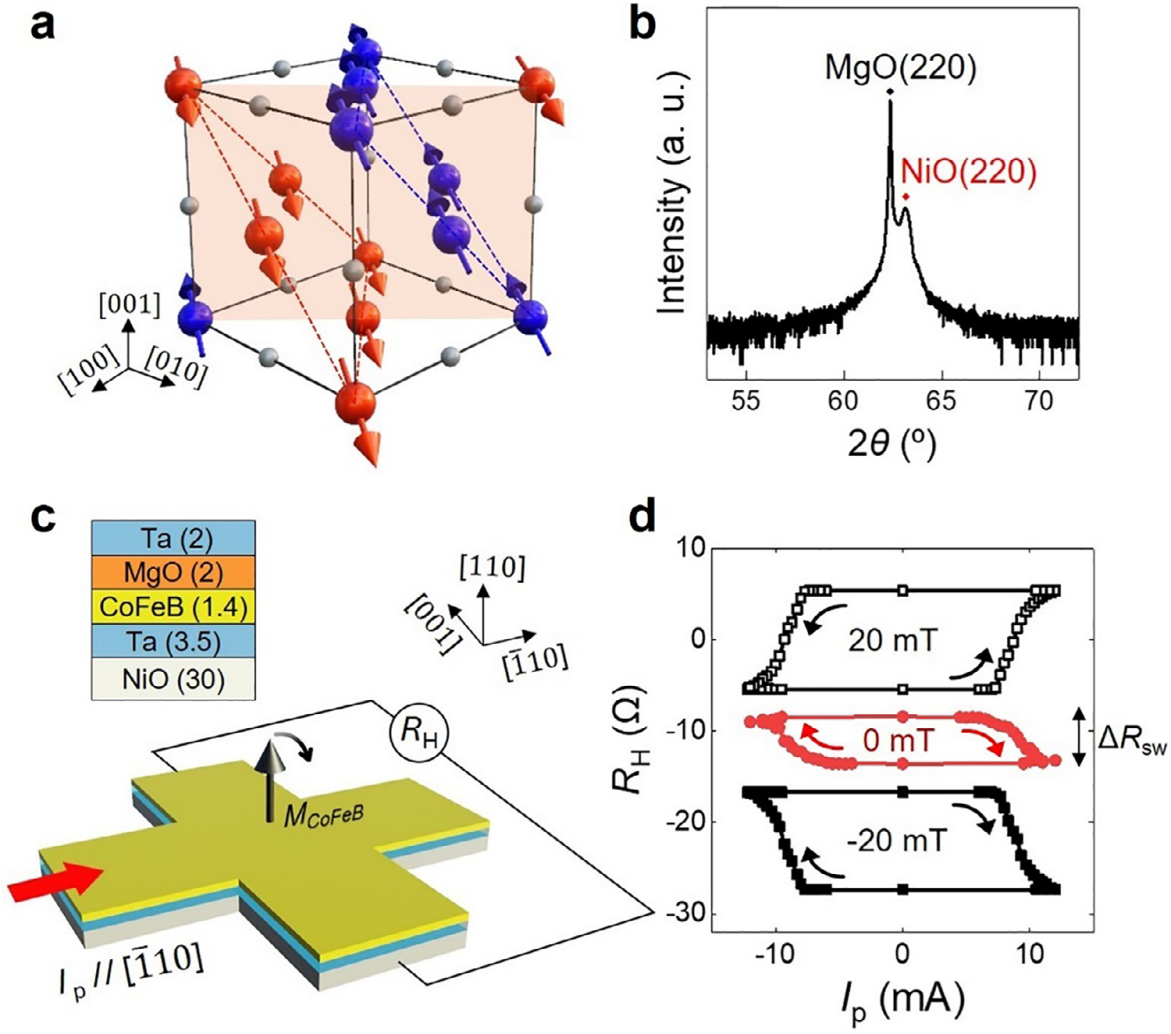
(a) Crystal and magnetic structures of NiO. (b) x‐ray diffraction (XRD) spectrum of a 30‐nm‐thick NiO (110) film grown on an MgO (110) substrate. (c) Schematic illustration of the current‐induced magnetization switching measurement for a NiO (30 nm)/Ta (3.5 nm)/CoFeB (1.4 nm)/MgO (2 nm) Hall bar device. A pulse current (*I*_p_) of 50‐µs width was applied along the [$\bar{1}10$] direction, inducing magnetization switching of the CoFeB layer (*M*_CoFeB_). (d) Current‐induced magnetization switching loops measured under various external magnetic fields (± 20 mT and 0 mT).

We first investigated SOT switching in an AFM‐based trilayer consisting of NiO (30 nm)/Ta (3.5 nm)/CoFeB (1.4 nm)/MgO (2 nm)/Ta (2 nm) structures, where the AFM insulator NiO was introduced as a spin current source. NiO features a rock salt structure as illustrated in Figure 1a, where Ni moments are ferromagnetically aligned within the {111} plane, while moments in neighboring {111} planes are antiferromagnetically coupled. In this study, we employed NiO (110) textured films grown on single‐crystalline MgO (110) substrates by RF magnetron sputtering, followed by annealing at 800°C to enhance crystallinity. Figure 1b presents the x‐ray diffraction (XRD) spectrum of a 30‐nm‐thick NiO (110), showing only the NiO (220) peak, which confirms heteroepitaxial growth on the MgO (110) plane. Due to the lattice mismatch with the MgO substrate, the NiO film experiences tensile strain, which induces an out‐of‐plane component of the AFM easy axis [37, 38]. On top of the NiO film, a Ta/CoFeB/MgO/Ta stack exhibiting perpendicular magnetic anisotropy was deposited by DC sputtering at room temperature (details are provided in the Experimental Section). Anomalous Hall resistance (*R*_H_) of the structure is shown in Supporting Information 1.

We then conducted current‐induced magnetization switching experiments using a Hall‐bar device with a 5 µm × 5 µm Hall cross (Figure 1c). A pulsed current (*I*_p_) with a pulse width of 50 µs was applied along the NiO $\left[\bar{1}10\right]$ direction, and the magnetization direction was monitored by measuring *R*_H_. Figure 1d presents the SOT switching results with different in‐plane magnetic fields. Under in‐plane magnetic fields of ±20 mT, the magnetization is fully switched by SOT with a switching current density of ∼4.0 × 10^7^ A/cm^2^, and the switching polarity was reversed upon reversal of the magnetic field. This field‐assisted SOT switching is attributed to the spin currents generated from the Ta layer with a negative spin Hall angle, consistent with previous studies [3, 39, 40]. Notably, SOT‐induced magnetization switching is also observed in the absence of an external magnetic field; however, the switching ratio, relative to the anomalous Hall resistance (Δ*R*_sw_/*R*_AHE_), remains approximately 50%. Although the switching is incomplete, field‐free magnetization switching is observed in the NiO‐based trilayer, indicating the presence of an unconventional SOT with an out‐of‐plane spin polarization, possibly originating from the NiO layer.

### 2.2 Angle‐Dependent SOT‐Induced Magnetization Switching

**FIGURE 2:**
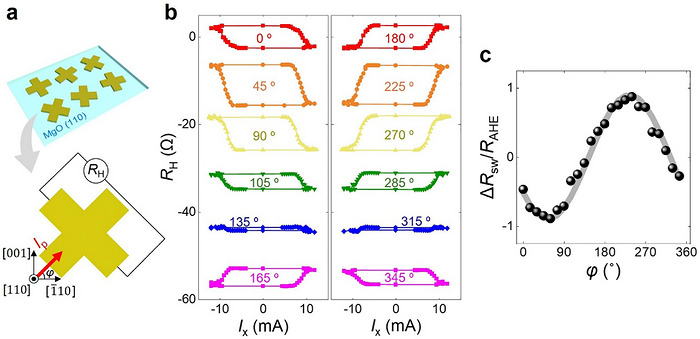
(a) Schematic illustration of Hall‐bar devices with different azimuthal angles *φ* relative to the $\left[\bar{1}10\right]$ direction in the NiO (110) plane. (b) Field‐free SOT‐induced magnetization switching loops measured for devices with various *φ* values. (c) Field‐free switching ratio extracted from (b), plotted as a function of *φ*. The gray line is a guide to the eye.

We next examined angle‐dependent SOT switching using Hall bar devices fabricated with various angles (*φ*) between the current direction and the NiO $\left[\bar{1}10\right]$ direction, as illustrated in Figure 2a. Note that all the fabricated Hall bar devices exhibit similar anomalous Hall resistances, coercivities, and anisotropy fields regardless of the *φ* values (Supporting Information 1). This indicates negligible device‐to‐device variations. Figure 2b presents field‐free SOT switching behavior for devices with different *φ* values. The switching loop at *φ* = 0° is identical to that shown in Figure 1d, with a switching ratio of around 50%. Remarkably, the switching ratio increases to nearly 100% at *φ* = 45° and decreases when *φ* exceeds 45°. In addition, the polarity of the field‐free switching reverses for *φ* values larger than 135°. We note that the field‐free switching behavior is independent of the initial magnetization state of the top CoFeB (Supporting Information 2). Figure 2c summarizes the switching ratio as a function of *φ*, demonstrating a cosine‐like dependence with a phase shift of approximately 50°. Note that the measurements were performed within a limited current range (± 12 mA) to minimize Joule‐heating‐induced artifacts, as higher current can significantly modify the switching behavior. Such effects hinder a direct correlation between switching current and SOT efficiency. Therefore, the switching ratio (Δ*R*_sw_/*R*_AHE_) is used as a practical indicator of the SOT‐induced switching efficiency [11, 41, 42, 43, 44, 45]. This *φ*‐dependent field‐free SOT switching ratio suggests that the unconventional SOT with out‐of‐plane spin polarization is governed by the crystallographic direction, likely associated with the Néel‐vector orientation of NiO. Note that in NiO (110), the AFM easy axis is generally expected to be oriented at an azimuthal angle of 55° relative to the $\left[\bar{1}10\right]$ direction, which is in close agreement with the observed phase shift. Consistently, angle‐dependent exchange bias and spin Hall magnetoresistance measurements confirm that the Néel vector in our samples is preferentially aligned close to 45° (Supporting Information 3 and 4).

To further confirm this angular dependence, we performed two complementary experiments. First, field‐free switching measurements were repeated using five devices with each *φ* value fabricated on the same substrate. As shown in Supporting Information 5, all device sets exhibited consistent angular dependence, demonstrating a common underlying anisotropy orientation in NiO films grown on the same substrate. Second, we fabricated devices on different substrates and measured their field‐free SOT switching behavior. While all device sets showed a similar cosine‐like angular dependence, the phase shift appeared at four distinct angles (Supporting Information 5). This phase shift can be understood as arising from the four symmetry‐allowed easy axis orientations in NiO(110), corresponding to the <121> family of directions. Accordingly, NiO films on different substrates can adopt one of these equivalent easy axes, leading to distinct phase shifts. Taken together, these angular dependences indicate that the unconventional SOT responsible for field‐free switching is governed by the AFM anisotropy of NiO(110). We note that such variation may need to be addressed for device applications, and the factors determining and controlling the Néel vector orientation are currently under investigation, including magnetic field annealing and the use of AFMs with uniaxial anisotropy.

We note that field‐free SOT switching has been reported previously in NiO/Pt/Co structures [46], where out‐of‐plane spin polarization was generated in the NiO/Pt bilayer. However, in those studies, the switching ratio was limited to approximately 50%, and no clear correlation between the switching behavior and the AFM anisotropy or Néel‐vector symmetry was demonstrated. In contrast, our results reveal nearly complete field‐free switching with a pronounced, symmetry‐governed angular dependence directly linked to the AFM anisotropy of NiO(110), clearly distinguishing this work from earlier reports.

### 2.3 Quantification of Unconventional Spin Currents

**FIGURE 3:**
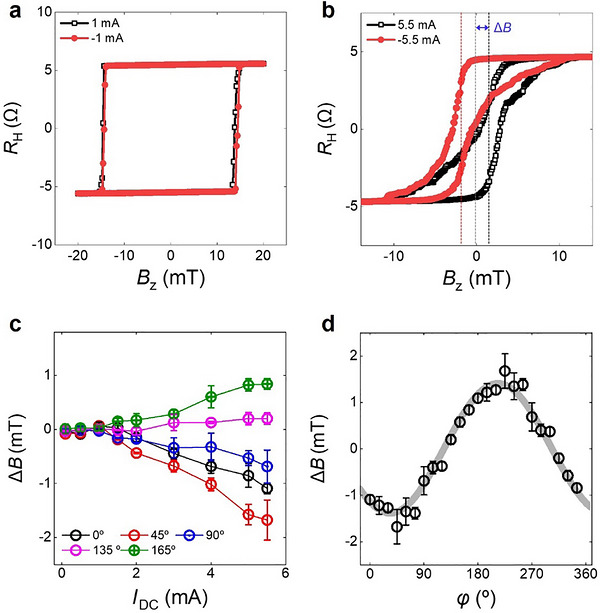
(a, b) Hysteresis loops of *R*_H_ vs. out‐of‐plane magnetic field (*B*_z_) measured for device with *φ* = 45° under d.c. currents of (a) |*I*_DC_| = 1 mA and (b) |*I*_DC_| = 5.5 mA. (c) Magnitude of the loop shift (Δ*B*) as a function of *I*_DC_ for the devices with various *φ* values. (d) *φ* dependence of Δ*B* measured at *I*_DC_ = 5.5 mA. The gray line is a guide to the eye.

We quantitatively examined unconventional spin currents with out‐of‐plane spin polarization by conducting anomalous Hall loop shift measurements [47]. Figure 3a,b show the *R*_H_ loops as a function of the out‐of‐plane magnetic field (*B*_z_) for a device with *φ* = 45°, measured under d.c. currents (*I*_DC_) of ± 1 mA and ± 5.5 mA, respectively. Note that no in‐plane magnetic field was applied during the measurements, ensuring that spin currents with in‐plane spin polarization did not contribute to the observed loop shifts. While the loop shifts are negligible at *I*_DC_ = ± 1 mA, they become pronounced at *I*_DC_ = ± 5.5 mA. Corresponding results for devices with *φ* = 0°, 90°, 135°, and 165° are shown in Supporting Information 6.

Figure 3c summarizes the magnitude of the loop shift (Δ*B*) as a function of *I*_DC_ for different *φ* values. Here, Δ*B* = [*B*_center_(–*I*_DC_)—*B*_center_(+*I*_DC_)]/2, where *B*_center_ is the center of the *R*_H_‐*B*_z_ loop, defined as the average of the up‐to‐down and down‐to‐up switching fields. The switching field was determined at the magnetic field at which the normalized anomalous Hall resistance crosses zero, within a tolerance range of ± 0.05. The Δ*B* values were averaged over at least three devices, and the error bars represent the standard deviation across devices. The results show that Δ*B* remains negligible for *I*_DC_ below 1 mA and then increases abruptly once *I*_DC_ exceeds a threshold current for all devices. This onset of the loop shift provides clear evidence for the presence of out‐of‐plane spin currents, which exert an anti‐damping torque on the perpendicular magnetization. The loop shift appears only when the current‐induced torque overcomes the intrinsic damping [20, 41, 48]. Figure 3d presents Δ*B* at *I*_DC_ = 5.5 mA as a function of *φ*, exhibiting a cos *φ* dependence. This indicates that the out‐of‐plane spin current reaches its maximum near *φ* = 45° and becomes nearly vanishing around *φ* = 135°. This angular dependence closely matches that of the current‐induced switching ratio (Figure 2c), confirming that the observed field‐free SOT switching originates from out‐of‐plane spin currents. Furthermore, the measured loop shift magnitude is Δ*B* ≈ 1.7 mT, corresponding to an effective field of |Δ*B*/*J*_DC_| ≈ 0.76 mT/(10^7^ A/cm^2^), which is comparable to values reported in previous studies [20, 41, 48].

### 2.4 Angular Dependence of Unconventional Spin Currents

**FIGURE 4:**
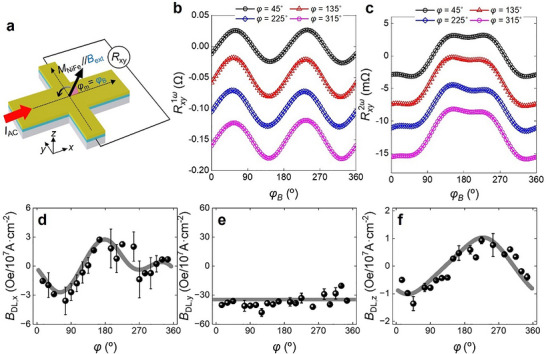
(a) Schematic illustration of the second harmonic measurement setup; the first and second harmonic Hall resistances ($R_{xy}^{1\omega }$ and $R_{xy}^{2\omega }$) are measured with an AC current *I*_AC_ while rotating the azimuthal angle of *B*_ext_ (φ_*B*_). (b, c) $R_{xy}^{1\omega }$ and $R_{xy}^{2\omega }\;$ as a function φ_*B*_ for the samples with different *φ* of 45°, 135°, 225°, and 315°. (d–f) Extracted SOT‐induced effective magnetic fields vs. *φ*, (d) *B*_**DL** *x*_, (e) *B*_**DL** *y*_, and (f) *B*_**DL** *z*_. The gray lines in (d–f) denote the calculated values from the micromagnetic simulations.

To further examine the spin currents generated in the NiO/Ta bilayer, we conducted in‐plane harmonic Hall measurements using NiO (30 nm)/Ta (3.5 nm)/NiFe (2 nm)/MgO (2 nm)/Ta (2 nm) heterostructures. In these measurements, we employed a NiFe layer with in‐plane magnetic anisotropy instead of the perpendicularly magnetized CoFeB used for the switching experiments. This allows for reliable measurements under a moderate external magnetic field (*B*_ext_) ranging from 0.1 to 1.2 T, which is sufficient to saturate the magnetization of NiFe (*M*_NiFe_), thereby ensuring a well‐defined in‐plane magnetization. Figure 4a schematically illustrates the measurement configuration, where the first and second harmonic Hall resistances ($R_{xy}^{1\omega }$ and $R_{xy}^{2\omega }$) are measured while rotating the azimuthal angle of *B*_ext_ (φ_B_). $R_{xy}^{1\omega }$ and $R_{xy}^{2\omega }$ are expressed as [49, 50]:

(1) $$R_{xy}^{1\omega }=R_{\mathrm{PHE}}\sin2\varphi_{B}$$

(2) $$\begin{array}{ccc} R_{xy}^{2\omega } & = & R_{\mathrm{AHE}}\frac{B_{\mathrm{DL}x}}{B_{\mathrm{eff}}}\sin\varphi_{B}-2R_{\mathrm{PHE}}\frac{B_{\mathrm{FL}x}}{B_{\mathrm{ext}}}\sin\varphi_{B}\cos2\varphi_{B}\\ & & \times \;\left(R_{\mathrm{AHE}}\frac{B_{\mathrm{DL}y}}{B_{\mathrm{eff}}}+R_{\nabla T}^{2\omega }\right)\cos\varphi_{B}-2R_{\mathrm{PHE}}\frac{B_{\mathrm{FL}y}+B_{\mathrm{Oe}}}{B_{\mathrm{ext}}}\\ & & \times \;\cos\varphi_{B}\cos2\varphi_{B}-2R_{\mathrm{PHE}}\frac{B_{\mathrm{DL}z}}{B_{\mathrm{ext}}}\cos2\varphi_{B}+R_{\mathrm{AHE}}\frac{B_{\mathrm{FL}z}}{B_{\mathrm{eff}}}, \end{array}$$

where *R*_PHE_ and *R*_AHE_ are the planar and anomalous Hall resistances, respectively; *B*_DL*y*_ (*B*_FL*y*_) are the damping‐like (field‐like) effective fields arising from conventional SOTs with *y*‐spin polarization, and *B*_DL*x*_ (*B*_FL*x*_) and *B*_DL*z*_ (*B*_FL*z*_) are the damping‐like (field‐like) effective fields induced by unconventional SOTs with *x‐* and *z*‐spin polarizations, respectively; *B*_Oe_ is the Oersted field, and *B*_eff_ is the effective magnetic field, *B*_eff_ = *B*_ext_  + *B*_ani_, where *B*_ani_ is the magnetic anisotropy field; $R_{\nabla T}^{2\omega }$ is the thermal contribution.

Figure 4b,c presents representative $R_{xy}^{1\omega }$ and $R_{xy}^{2\omega }$ data as a function of φ_B_ for the devices with *φ* = 45°, 135°, 225°, and 315°, measured at *B*_ext_ = 150 mT. Data for devices with other *φ* values is provided in Supporting Information 7. By fitting the $R_{xy}^{1\omega }$ and $R_{xy}^{2\omega }$ curves using Equations (1) and (2), we extracted the effective magnetic fields for each device at different *φ* values. Figure 4d–f summarizes the obtained *B*_DL*x*_, *B*_DL*y*_, and *B*_DL*z*_ as a function of *φ*, respectively. The corresponding field‐like effective fields are presented in Figure S15 of Supporting Information 7. These results reveal that all three damping‐like effective magnetic fields exhibit finite magnitudes with distinct angular dependencies. The magnitude of *B*_DL*y*_ remains nearly constant regardless of *φ* (Figure 4e), indicating that it is primarily dominated by conventional SOT due to the spin Hall effect in the Ta layer. Remarkably, the sizable *B*_DL*x*_ and *B*_DL*z*_ components exhibit pronounced angular dependences, providing direct evidence for the presence of unconventional SOTs in the AFM‐based trilayers, as these components are absent in control structures without the NiO layer (Supporting Information 8). Note that the effective fields obtained from the harmonic Hall measurement (Figure 4f) are smaller than those extracted from the anomalous Hall loop‐shift method (Figure 3d). This difference likely arises because the loop‐shift measurement was performed at higher current densities, resulting in stronger Joule heating. This reduces the coercive field and can lead to an overestimation of the effective field. In addition, the two methods probe different magnetic energy scales, which can lead to different extracted effective fields: the loop‐shift measurement involves the domain‐wall depinning field, whereas the harmonic Hall measurement reflects the linear response of a quasi‐single‐domain state governed by a much larger perpendicular magnetic anisotropy field.

To account for the unconventional spin currents observed in the AFM‐based trilayers, we consider a possible mechanism based on AFM spin dynamics, examined using micromagnetic simulations (see details in Supporting Information 9). In this scenario, when a charge current (**J**_c_) is applied to an AFM/NM/FM trilayer, the spin Hall current (**J**_s_) generated in the NM layer can excite spin dynamics in the bottom AFM layer [35, 36]. These AFM dynamics can, in turn, generate spin‐pumping‐induced spin currents (**J**_sp_) that flow back through the NM layer and exert a spin torque on the top FM layer. **J**_sp_ is expressed as $J_{\mathrm{sp}}=g_{r}^{↑↓}\;\hbar /8\pi \sum \left(m_{1}\times \partial_{t}m_{1}+m_{2}\times \partial_{t}m_{2}\right)$, where $g_{r}^{↑↓}$ is the spin mixing conductance at the AFM/NM interface, and **m**_*i*_ denote the unit vectors of the two AFM sublattices [35]. Under sufficiently strong spin torques, the AFM moments can enter a steady‐state precession regime [51, 52], leading to continuous spin pumping into the NM layer. We apply this framework to our NiO(110)/Ta/CoFeB trilayer structure.

Using this model, we calculated the effective spin Hall angles of the spin pumping‐induced spin current components (θ_sp,*i*_), defined as 2*eJ*_sp,*i*_/ℏ*J*_c_, where *i* = *x, y*, and *z*. Here, we assume the easy axis of NiO is aligned along the [211] direction; results for three other equivalent easy axes of NiO are presented in Supporting Information 9. The calculated θ_sp,*i*_ was then converted into the corresponding damping‐like effective fields, which are plotted as solid lines in Figure 4d–f. These simulated angular dependences show qualitative agreement with the experimentally observed *φ*‐dependent variations of *B*_DL*x*_ and *B*_DL*z*_. In particular, *B*_DL*z*_ approaches zero near *φ* = 135° and 315°, consistent with the *φ*‐dependent field‐free SOT switching ratio shown in Figure 2c. Taken together, these results indicate that AFM spin dynamics‐induced spin pumping provides a plausible mechanism that captures the key angular features of the unconventional spin currents observed in our AFM‐based trilayers. While other contributions cannot be fully excluded, the consistency between the simulations and experiments suggests that AFM spin dynamics play an important role in generating unconventional spin currents and the associated field‐free SOT switching.

### 2.5 SOT Switching Results in Control Samples

**FIGURE 5:**
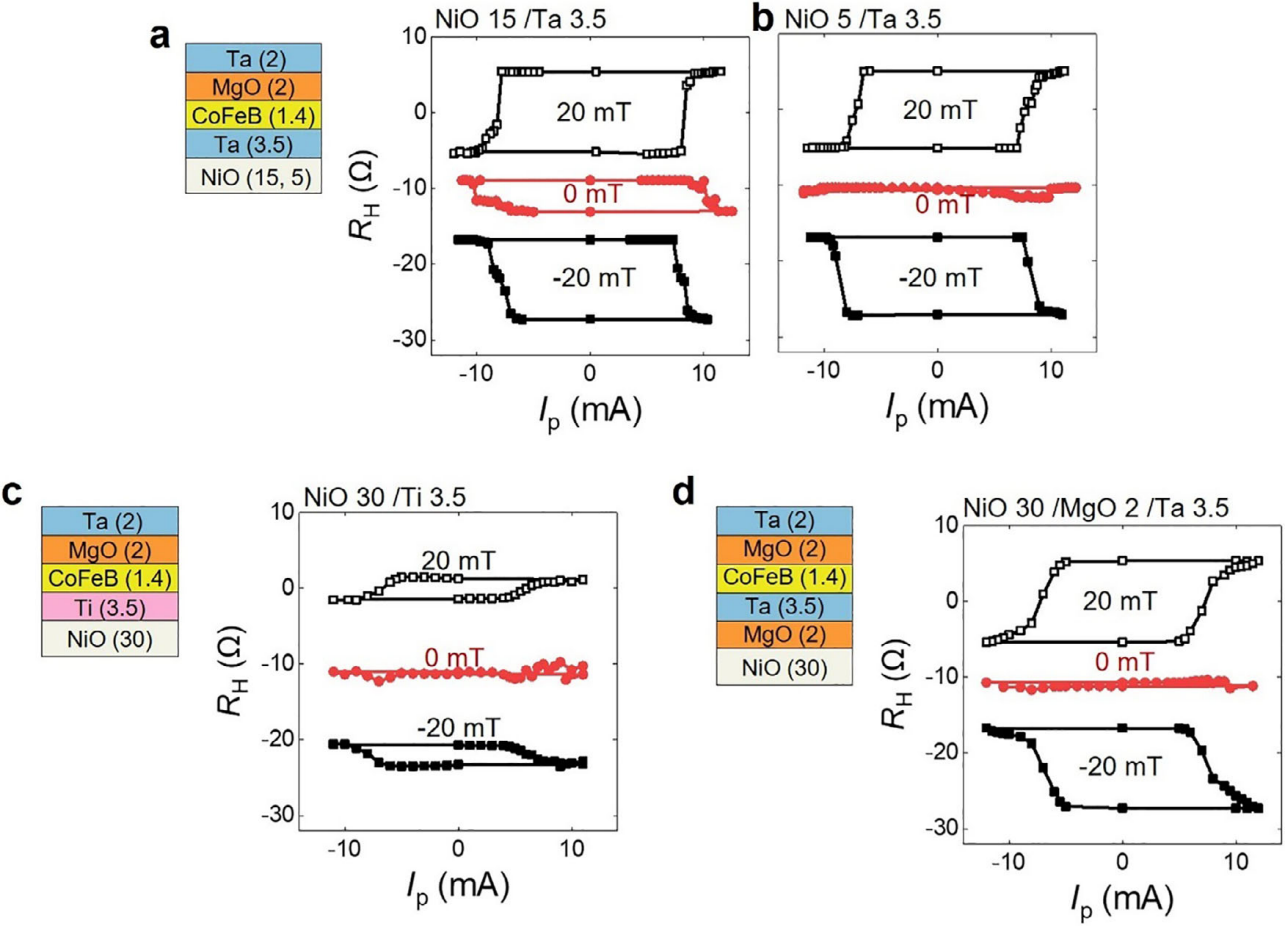
(a, b) Current‐induced magnetization switching loops of NiO (*t*_NiO_​)/Ta (3.5 nm)/CoFeB (1.4 nm)/MgO structure with (a) *t*_NiO_​ = 15 nm and (b) 5 nm. (c) Current‐induced magnetization switching loops of a NiO (30 nm)/Ti (3.5 nm)/CoFeB (1.4 nm)/MgO structure. (d) Current‐induced magnetization switching loops of a NiO (30 nm)/MgO (2 nm)/Ta (3.5 nm)/CoFeB (1.4 nm)/MgO structure.

To further support the proposed scenario, we performed two types of control experiments that independently probe two key ingredients for AFM spin pumping: (i) the AFM ordering and (ii) the efficiency of spin‐current injection into the AFM layer. First, to examine the role of the AFM ordering, we investigated samples with different NiO thicknesses (*t*_NiO_) of 15 and 5 nm. Figure 5a,b presents the current‐induced switching loops measured with and without *B_x_*, where pulsed currents were applied along the $\left[\bar{1}10\right]$ direction (*φ* = 0°). When applying a *B_x_* of ±20 mT, current‐induced magnetization switching is observed for both samples, consistent with the behavior for the thicker NiO sample shown in Figure 1d. In contrast, the field‐free SOT switching ratio strongly depends on *t*_NiO_; it is significantly reduced for *t*_NiO_ = 15 nm (Figure 5a) and is entirely suppressed for *t*_NiO_ = 5 nm (Figure 5b). Because AFM ordering in NiO is known to strengthen with increasing NiO thickness (Supporting Information 10) [53, 54], these results indicate that robust AFM ordering is a prerequisite for the emergence of unconventional out‐of‐plane SOT.

Second, to assess the role of the NiO/Ta interface in spin‐current injection, we examined two additional control samples: (i) NiO (30 nm)/Ti (3.5 nm)/CoFeB (1.4 nm)/MgO, where Ti with a negligible spin Hall angle replaces Ta, and (ii) NiO (30 nm)/MgO (2 nm)/Ta (3.5 nm)/CoFeB (1.4 nm)/MgO, where an insulating MgO layer is inserted at the NiO/Ta interface. Figure 5c presents that in the NiO/Ti/CoFeB/MgO sample, even under *B_x_* = ± 40 mT, the SOT switching ratio is significantly reduced to 30%, and field‐free SOT switching is absent. This is attributed to the insufficient spin currents generated from Ti [20], which cannot excite AFM spin dynamics. Figure 5d presents the results for the NiO/MgO/Ta/CoFeB/MgO sample, where full SOT switching is achieved under a magnetic field, but field‐free switching is suppressed due to the MgO interlayer blocking spin current transmission into the AFM layer.

Furthermore, an orbital contribution may be present in our samples, as the orbital Rashba‐Edelstein effect can arise at NiO/Ta interfaces [55, 56], and orbital current generation and conversion are known to depend on the crystalline structure [57, 58]. However, if this mechanism were dominant, similar behavior should also be observed in the NiO/Ti control sample, since Ti is a widely used orbital source material [59, 60]. The absence of field‐free switching in the NiO/Ti sample therefore suggests that the orbital currents are unlikely to be the dominant origin of the observed effect in our system.

These control experiments demonstrate that both robust AFM ordering and efficient spin‐current injection into the AFM layer are essential for realizing field‐free SOT switching. This conclusion is consistent with the micromagnetic simulations, which suggest that unconventional SOT with out‐of‐plane spin polarization arises from the AFM spin dynamics.

## 3 Conclusions

In this work, we have demonstrated that AFM‐based trilayers can generate unconventional spin currents and the associated SOTs, with the AFM layer acting as an active spin current source. In NiO(110)/Ta/CoFeB trilayers, the field‐free switching efficiency exhibits a pronounced angular dependence, reaching nearly complete switching when the current is applied transverse to the AFM easy axis. These results indicate that the generated spin currents are governed by the Néel‐vector orientation relative to the current direction. Furthermore, harmonic Hall measurements reveal the coexistence of spin current components with both in‐plane and out‐of‐plane spin polarizations, each exhibiting distinct angular dependences. These observations are in good agreement with a scenario in which AFM‐driven spin dynamics provide a plausible mechanism for the unconventional spin currents. This interpretation is further supported by control experiments, which demonstrate that robust AFM ordering and efficient spin‐current injection into the AFM layer are essential for realizing field‐free SOT switching. Our findings establish AFM‐based trilayers as promising platforms for energy‐efficient field‐free SOT applications, opening new opportunities for AFM Néel‐vector‐controlled spintronic devices.

## 4 Methods

### 4.1 Sample Preparation

Thin film heterostructures were grown on MgO (110) substrates using RF and DC magnetron sputtering. Three different stacking structures were fabricated: (i) MgO (110) sub./NiO (5, 15, 30)/Ta (3.5)/CoFeB (1.4)/MgO (2)/Ta (2), (ii) MgO (110) sub./NiO (30)/Ti (3.5)/CoFeB (1.4)/MgO (2)/Ta (2), (iii) MgO (110) sub./NiO (30)/MgO (2)/Ta (3.5)/CoFeB (1.4)/MgO (2)/Ta (2), where the numbers in parentheses indicate the layer thickness in nanometers. The NiO layers were deposited using RF magnetron sputtering at a power of 50 W, a working pressure of 5 mTorr, and an Ar gas flow rate of 22.5 sccm. To enhance crystallinity, the samples were annealed at 800°C after NiO deposition. The subsequent multilayers were sputtered at room temperature under a base pressure of 3 × 10^−8 ^Torr. The working pressure was maintained at 3 mTorr for metallic layers and 10 mTorr for MgO layers. During this process, all metallic layers were deposited using DC magnetron sputtering with a power of 30 W, while the MgO layers were deposited using RF magnetron sputtering at a power of 150 W. After deposition, the samples were annealed at 215°C to induce perpendicular magnetic anisotropy in the CoFeB layer. The thin films were patterned into Hall cross devices with a Hall cross area of 5 × 5 µm^2^ and a length of 15 µm, using photolithography and Ar ion milling.

### 4.2 Electrical Measurements

Spin‐orbit torque (SOT) switching was examined by applying a current pulse (*I*_p_) of 50‐µs pulse width. The magnetization state was monitored after each *I*_p_ through measurement of the anomalous Hall resistance (*R*_H_) with a DC current of 0.1 mA. For harmonic Hall measurements, two lock‐in amplifiers were employed to simultaneously detect the first and second harmonic Hall resistances ($R_{xy}^{1\omega }$ and $R_{xy}^{2\omega }$). An AC current with a frequency of 13.71 Hz was supplied, and the sample was rotated while the external magnetic field remained fixed. All measurements were carried out at room temperature.

## Author Contributions

**Geunwoo Kim**: investigation. **Jong‐Ryul Jeong**: investigation. **Hyeong‐Joo Seo**: investigation, methodology, formal analysis, validation, writing – original draft. **Jong Hoon Jung**: investigation, methodology. **Byong‐Guk Park**: investigation, conceptualization, formal analysis, project administration, supervision, writing – original draft. **Soojung Kim**: investigation, methodology. **Dong‐hyeon Han**: investigation. **Kyung‐Jin Lee**: investigation, formal analysis, writing – review and editing. **Seok‐Jong Kim**: investigation, formal analysis. **Phuoc Cao Van**: investigation, methodology. **Younghun Jo**: investigation, methodology. **Chun‐Yeol You**: investigation, methodology. **Hong‐Cuong Truong**: investigation.

## Conflicts of Interest

The authors declare no conflicts of interest.

## Supporting information




**Supporting File**: advs76444‐sup‐0001‐SuppMat.docx.

## Data Availability

The data that support the findings of this study are available on request from the corresponding author. The data are not publicly available due to privacy or ethical restrictions.
