# Supplementary material for: Néel‐Vector‐Dependent Unconventional Spin‐Orbit Torque for Deterministic Field‐Free Switching in NiO (110)‐Based Trilayers
Source: Adv Sci (Weinh). 2026 Jul 9;13(54):e76444. doi: 10.1002/advs.76444 (PMC13348334; doi:10.1002/advs.76444)
Supplement: Supplementary file 1 [file ADVS-13-e76444-s001.docx]

Supporting Information

**Néel-vector-dependent unconventional spin-orbit torque for deterministic field-free switching in NiO (110)-based trilayers**

Hyeong-Joo Seo, Seok-Jong Kim, Phuoc Cao Van, Hong-Cuong Truong, Dong-hyeon Han, Geunwoo Kim, Younghun Jo, Soojung Kim, Chun-Yeol You, Jong-Ryul Jeong, Kyung-Jin Lee, and Byong-Guk Park*

**Supporting Information 1. Anomalous Hall resistances of NiO/Ta/CoFeB samples**

**Supporting Information 2. Field-free SOT switching under initialization conditions**

**Supporting information 3. Angle-dependent exchange bias in NiO(110)/CoFeB bilayers**

**Supporting information 4. Magnetic-field-dependent spin Hall magnetoresistance in NiO(110)/CoFeB bilayers**

**Supporting Information 5. Angular dependence of field-free SOT switching**

**Supporting Information 6. Anomalous Hall loop shift measurements**

**Supporting Information 7. Spin-orbit torque measurements in NiO/Ta/NiFe sample**

**Supporting Information 8. Spin-orbit torque measurements in Ta/NiFe reference sample**

**Supporting Information 9. Calculation of effective spin Hall angle from spin pumping for *x*-, and *y*- components and other easy axes**

**Supporting Information 10. NiO Thickness dependence of antiferromagnetic ordering**

**Supporting Information 1. Anomalous Hall resistances of NiO/Ta/CoFeB samples**

We measured anomalous Hall resistance (*R*_H_) as a function of out-of-plane magnetic field (*B_z_*) of a NiO (30 nm)/Ta(3.5 nm)/CoFeB(1.4 nm)/MgO(2 nm) structure. Figure S1a shows the *R*_H_ versus *B_z_* curve measured with a current applied along the [$\bar{1}10$] direction of the NiO (110) plane, demonstrating perpendicular magnetic anisotropy of the CoFeB layer with *R*_AHE_ ≈ 10.6 Ω. Figure S1b presents the anomalous Hall loops for devices with different azimuthal angles (*φ*). The curves exhibit similar loop shapes and nearly identical *R*_AHE_ values across different *φ*. Figure S1c shows the coercive field (*B*_c_) averaged over six devices at each angle. The average *B*_c_ remains nearly constant (~25 mT) regardless of *φ.*

Furthermore, we measured the magnetic anisotropy of devices with different *φ* values. Figure S2a shows the normalized anomalous Hall resistance (*R*_H_) as a function of the external in-plane magnetic field (*B*_ext_), revealing that nearly identical *R*_H_–*B*_ext_ curves across all devices. Using the generalized Sucksmith-Thompson (GST) method, we extracted the anisotropy field (*B*_k_), exhibiting no considerable *φ*-dependence (Figure S2b). Note that minor variations are likely due to fabrication-induced differences.

Figure S1. a) Anomalous Hall resistance (*R*_H_) versus out-of-plane magnetic field (*B_z_*) in the NiO (30 nm)/Ta(3.5 nm)/CoFeB(1.4 nm)/MgO(2 nm) sample. b) Anomalous Hall resistance loops for the samples with various azimuthal angles (*φ*). c) Averaged *B*_c_ as a function of *φ.*

Figure S2. a) Normalized anomalous Hall resistance (*R*_H_/*R*_H,sat_) as a function of in-plane magnetic field *B*_ext_ for devices with different azimuthal angles *φ*. b) Extracted *B*_k_ as a function of *φ*.

**Supporting Information 2. Field-free SOT switching under initialization conditions**

We performed additional switching measurements under the +*z* and −*z* initialization conditions. Figure S3a shows the field-free magnetization switching curve for the device with *φ* = 0° under +*z* initialization (the same sample as in Figure 1d). The current is swept from +12 mA to −12 mA and back to +12 mA, forming a minor switching loop. Upon the reverse current sweep, the magnetization does not fully return to the initial state, resulting in incomplete switching with a switching ratio of approximately 50 %. We attribute this to insufficient spin currents with out-of-plane spin polarization combined with Joule heating effect. Figure S3b shows the corresponding curve under −*z* initialization. The resulting loop is nearly identical to that in Figure S3a, indicating that the switching behavior is independent of the initial magnetization state.

We further performed the same experiments for the device with *φ* = 45°, where full switching is achieved. Figures S3c and S3d show the switching curves obtained after +*z* and −*z* initialization, respectively, confirming that the switching behavior remains independent of the initialization condition.

**Figure S3.** a, b) Field-free current-induced switching curves for the device with *φ* = 0°, measured after (a) +*z* and (b) −*z* initialization c, d) Field-free current-induced switching curves for the device with *φ* = 45°, measured after (c) +*z* and (d) −*z* initialization.

**Supporting information 3. Angle-dependent exchange bias in NiO(110)/CoFeB bilayers**

We investigated angle-dependent exchange bias in a NiO(30 nm)/CoFeB(3 nm) bilayer to probe the Néel vector orientation. The exchange bias field is expected to be largest when the magnetic field is applied along the Néel vector direction. Figures S4a-d show the magnetization curves measured at 40 K after field cooling from 390 K under a magnetic field of ±7 T for different in-plane angles ($\varphi_{B}$ = 0°, 45°, 90°, and 135°). The extracted exchange bias field (*B*_ex_) is summarized in Figure S4e. Note that the error bars in Figure S4e represent the standard deviation of the exchange-bias fields obtained from three independent measurements. We find that *B*_ex_ reaches a maximum (~9.2 mT) at $\varphi_{B}$ = 45° and a minimum (~2.7 mT) at $\varphi_{B}$ = 135°, indicating that the Néel vector in our NiO(110) film is preferentially aligned to close to *φ* = 45°.

Figure S4. a-d) Magnetic hysteresis loops measured at 40 K for (a) $\boldsymbol{\varphi}_{\boldsymbol{B}}$ = 0°, (b) 45°, (c) 90°, and (d) 135°. e) Extracted *B*_ex_ as a function of $\boldsymbol{\varphi}_{\boldsymbol{B}}$. The error bars represent the standard deviation of the exchange-bias fields obtained from three independent measurements performed under identical field-cooling conditions.

**Supporting Information 4. Magnetic-field-dependent spin Hall magnetoresistance in NiO(110)/Pt bilayers**

We performed magnetic-field-dependent transport measurements on a NiO(30 nm)/Pt(4 nm) bilayer to independently probe the Néel vector orientation via spin Hall magnetoresistance. The measurements were carried out on a Hall-bar device with *φ* = 45° relative to the crystallographic $[\bar{1}10]$ direction of NiO(110), corresponding to the configuration that exhibits the largest unconventional SOT in the main text. The longitudinal resistance ($R_{xx}$) was measured while sweeping an external magnetic field ($B_{\mathrm{ext}}$) for different azimuthal angles ($\varphi_{B}$= 0°, 45°, 90°, and 135°) as schematically illustrated in Figure S5a. As shown in Figure S5b, for $\varphi_{B}$ = 0°, a pronounced anomaly appears near $B_{\mathrm{ext}}\approx5.5$T, whereas $R_{xx}$ evolves smoothly for other field orientations (Figures S5c-e). In antiferromagnetic NiO, a spin-flop transition–an abrupt reorientation of the Néel vector–occurs when the magnetic field is applied along the easy axis ^[S1]^. Therefore, the observed anomaly for $\varphi_{B}$ = 0° clearly indicates that the Néel vector is aligned along this direction, i.e., parallel to the current direction in this device geometry (*φ* = 45°).

Figure S5. a) Schematic of spin Hall magnetoresistance measurement on the NiO/Pt Hall-bar device. b-e) The longitudinal resistance ($\boldsymbol{R}_{\boldsymbol{xx}}$) as a function of an external magnetic field ($\boldsymbol{B}_{\mathbf{ext}}$) for different azimuthal angles ($\boldsymbol{\varphi}_{\boldsymbol{B}}$), (b) $\boldsymbol{\varphi}_{\boldsymbol{B}}$= 0°, (c) 45°, (d) 90°, and (e) 135°.

To further verify the reliability of the spin Hall magnetoresistance measurements, we performed the additional measurements: (i) repeated measurements on the same device to verify reproducibility and (ii) temperature-dependent measurements to examine the evolution of the spin-flop field.

First, Figure S6 show four independent measurements performed on the same device with *φ* = 45° under the identical condition, where the magnetic field was applied along $\varphi_{B}$ = 0°. The characteristic anomaly associated with the spin-flop transition is consistently observed near $B_{\mathrm{SF}}\approx5.5$ T in all measurements, confirming the reproducibility of the spin-flop anomaly in our NiO sample.

Second, we investigated the temperature dependence of the spin-flop transition. Figures S7a and S7b show the results obtained at 200 K, while Figures S7c,d show those obtained at 100 K. In all cases, a clear spin-flop anomaly was observed, enabling reliable extraction of the spin-flop field. Figure S8 summarizes the extracted spin-flop fields field ($B_{\mathrm{SF}}$) as a function of measurement temperature. The spin-flop field gradually increases from 5.57 T at 300 K to 6.15 T at 100 K, consistent with the expected strengthening of the antiferromagnetic anisotropy at lower temperatures ^[S1]^. Note that the error bars represent the standard deviation obtained from repeated measurements.

Figure S6. Reproducibility of the spin-flop transition. (a–d) Four independent measurements performed on the same NiO(30 nm)/Pt(4 nm) Hall-bar device under the identical experimental conditions.

**Figure S7.** **Temperature-dependent spin Hall magnetoresistance of the NiO(30 nm)/Pt(4 nm) Hall-bar device**. (a,b) Two independent measurements at 200 K. (c,d) Two independent measurements at 100 K.

**Figure S8.** Extracted spin-flop field ($B_{\mathrm{SF}}$) as a function of temperature. Error bars represent the standard deviation obtained from repeated measurements.

**Supporting Information 5. Angular dependence of field-free SOT switching**

To verify the reproducibility of the angle-dependent field-free SOT switching, we repeated the measurements with five additional devices of the NiO (30 nm)/Ta(3.5 nm)/CoFeB(1.4 nm)/MgO(2 nm) structure, fabricated on the same MgO (110) substrate. The switching ratio (Δ*R*_sw_/*R*_AHE_) was evaluated for NiO (30 nm)/Ta(3.5 nm)/CoFeB(1.4 nm)/MgO(2 nm)/Ta(2 nm) samples with varying *φ*. Figure S9 presents the switching ratio of the devices. The results demonstrate that all devices exhibit a consistent angular dependence, confirming the reproducibility of the observed switching polarity and switching ratio.

We further fabricated additional devices grown on different MgO (110) substrates. Figure S10a-d presents representative field-free SOT switching loops with different *φ*-angle dependencies among the multiple samples. Figure S10e shows the extracted switching ratio as a function of *φ* for the samples, demonstrating that all devices exhibit a cosine-like dependence, with a phase shift of ~90°. These four distinct phase shifts are consistent with four possible easy axes of the NiO (110) texture film, and their azimuthal angles are 55°, 125°, 235°, and 305° relative to the $[\bar{1}10]$ axis, as illustrated in Figure S11.

**Figure S9.** Switching ratio (Δ*R*_sw_/*R*_AHE_) of multiple devices fabricated on the same MgO (110) substrate. The line are guides to the eye.

**Figure S10**. a-d) Field-free SOT switching loops of four different samples of the NiO (30 nm)/Ta(3.5 nm)/CoFeB(1.4 nm)/MgO(2 nm) structures with different *φ*’s. e) Switching ratio (Δ*R*_sw_/*R*_AHE_) as a function of *φ*. The lines guide for the eye.

Figure S11. a-d) Possible magnetization configurations of the NiO (110) texture film, with four different Néel vector configurations; (a) 55°,(b) 125°,(c) 235°, and (d) 305°, relative to the [$\bar{\mathbf{1}}\mathbf{10}$] direction.

**Supporting Information 6. Anomalous Hall loop shift measurements**

We provide additional *R*_H_ –*B_z_* loops for devices with *φ* = 0°, 90°, 135°, and 165°, which were not included in the main text. As shown in Figure S12, negligible loop shifts are observed at *I*_DC_ = ±1 mA, while clear shifts appear at *I*_DC_ = ±5.5 mA, consistent with the trend shown for the *φ* = 45° device in the main text. The corresponding Δ*B* values as a function of *I*_DC_ are plotted in Figure 3. These results confirm that the abrupt onset of loop shift above ~1 mA is consistently observed across different *φ* values.

Figure S12. *R*_H_ as a function of *B_z_* measured under *I*_DC_ = ±1 mA (left column) and ±5.5 mA (right column) for devices with *φ* = 0°, 90°, 135°, and 165°.

Supporting Information 7. Spin-orbit torque measurements in NiO/Ta/NiFe sample

Figure S13 presents the $R_{xy}^{2\omega}$ as a function of $\varphi_{B}$ of samples with various *φ*’s, measured under an external magnetic field of 150 mT. To obtain the SOT effective fields, we separate $\cos\varphi_{B},$ $\cos\varphi_{B}\cos2\varphi_{B}$, $\sin\varphi_{B}$, $\sin\varphi_{B}\cos2\varphi_{B}$, and $\cos2\varphi_{B}$ components from the $R_{xy}^{2\omega}$ data using Equation (2) of the main text, which corresponding to the ${R_{\mathrm{DL}y}^{2\omega}}/{R_{\mathrm{AHE}}}$, ${R_{\mathrm{FL}y}^{2\omega}}/{R_{\mathrm{PHE}}}$, ${R_{\mathrm{DL}x}^{2\omega}}/{R_{\mathrm{AHE}}}$, ${R_{\mathrm{FL}x}^{2\omega}}/{R_{\mathrm{PHE}}}$, ${R_{\mathrm{DL}z}^{2\omega}}/{R_{\mathrm{PHE}}}$, respectively. Figure S14 shows an example of this separation for the device with *φ* = 45°. Then, we plotted each component as a function of *B*_eff_ or *B*_ext_ (Figures S15a-e), where the slopes correspond to the effective SOT fields. Figure S15f shows the extracted $B_{\mathrm{FL}x}^{2\omega}$ and $B_{\mathrm{FL}y}^{2\omega}$ as a function of *φ*.

Figure S13. Second harmonic Hall resistance ($\boldsymbol{R}_{\boldsymbol{xy}}^{\mathbf{2}\boldsymbol{\omega}}$) as a function of $\boldsymbol{\varphi}_{\boldsymbol{B}}$ under an external magnetic field of 150 mT for the samples with various *φ*’s.

Figure S14. a) $\boldsymbol{R}_{\boldsymbol{xy}}^{\mathbf{2}\boldsymbol{\omega}}$ for the device with *φ* = 45°. Black dots represent experimental data. b-f) each component of $\boldsymbol{R}_{\boldsymbol{xy}}^{\mathbf{2}\boldsymbol{\omega}}$: (b) $\cos\boldsymbol{\varphi}_{\mathbf{B}}$, (c) $\cos\boldsymbol{\varphi}_{\mathbf{B}}\cos\boldsymbol{2}\boldsymbol{\varphi}_{\mathbf{B}}$, (d) $\sin\boldsymbol{\varphi}_{\mathbf{B}}$, (e) $\sin\boldsymbol{\varphi}_{\mathbf{B}}\cos\boldsymbol{2}\boldsymbol{\varphi}_{\mathbf{B}}$, and (f) $\cos\boldsymbol{2}\boldsymbol{\varphi}_{\mathbf{B}}$ as a function of $\boldsymbol{\varphi}_{\boldsymbol{B}}$.

**Figure S15.** a-f) Each $\varphi_{B}$-dependent component plotted as a function of 1/*B*_eff_ (or 1/*B*_ext_), (a) ${R_{\mathrm{DL}x}^{2\omega}}/{R_{\mathrm{AHE}}}$ versus 1/*B*_eff_, (b) ${R_{\mathrm{DL}y}^{2\omega}}/{R_{\mathrm{AHE}}}$ versus 1/*B*_eff_, (c) ${R_{\mathrm{DL}z}^{2\omega}}/{R_{\mathrm{PHE}}}$ versus 1/*B*_ext_, (d) ${R_{\mathrm{FL}x}^{2\omega}}/{R_{\mathrm{AHE}}}$ versus 1/*B*_eff_, (e) ${R_{\mathrm{FL}y}^{2\omega}}/{R_{\mathrm{AHE}}}$ versus 1/*B*_eff_, and (f) Extracted $B_{\mathrm{FL}x}$, $B_{\mathrm{FL}y}$ as a function of $\varphi$.

**Supporting Information 8. Spin-orbit torque measurements in Ta/NiFe reference sample**

Figure S16 shows the $R_{xy}^{2\omega}$ as a function of $\varphi_{B}$ for the Ta/NiFe reference sample without NiO layer, measured under an external magnetic field of 150 mT. Representative data are presented for devices with *φ* = 0, 45 and 90°.

Using Equation (2) in the main text, the $\cos\varphi_{B}$ and $\cos2\varphi_{B}$ components were separated from the $R_{xy}^{2\omega}$ data, corresponding to ${R_{\mathrm{DL}y}^{2\omega}}/{R_{\mathrm{AHE}}}$ and ${R_{\mathrm{DL}z}^{2\omega}}/{R_{\mathrm{PHE}}}$, respectively. Figure S17 shows the fitting results for these components.

The extracted ${R_{\mathrm{DL}y}^{2\omega}}/{R_{\mathrm{AHE}}}$ exhibits a finite value, whereas ${R_{\mathrm{DL}z}^{2\omega}}/{R_{\mathrm{PHE}}}$ remains negligible within the measurement accuracy for all measured *φ*.

Figure S16. Second harmonic Hall resistance ($\boldsymbol{R}_{\boldsymbol{xy}}^{\mathbf{2}\boldsymbol{\omega}}$) for Ta/NiFe sample as a function of $\boldsymbol{\varphi}_{\boldsymbol{B}}$ under an external magnetic field of 150 mT for the samples with various *φ*’s.

**Figure S17.** Each $\varphi_{B}$-dependent component plotted as a function of 1/*B*_eff_ (or 1/*B*_ext_), ${R_{\mathrm{DL}y}^{2\omega}}/{R_{\mathrm{AHE}}}$ versus 1/*B*_eff_, and ${R_{\mathrm{DL}z}^{2\omega}}/{R_{\mathrm{PHE}}}$ versus 1/*B*_ext_.

**Supporting Information 9. Calculation of effective spin Hall angles based on AFM spin pumping**

We performed atomistic model simulations based on the Landau-Lifshitz-Gilbert (LLG) equation for a one-dimensional antiferromagnetic system consisting of of non-magnet (NM)/antiferromagnet (AFM) structures:

$\partial_{t}\mathbf{m}_{i}=-\gamma\mathbf{m}_{i}\times\mathbf{H}_{\mathrm{eff},i}+\alpha\mathbf{m}_{i}\times\partial_{t}\mathbf{m}_{i}+\gamma c_{j}\mathbf{m}_{i}\boldsymbol{\times}\left( \mathbf{m}_{i}\boldsymbol{\times}\hat{\mathbf{y}} \right)$*.*

This equation incorporates precession torque, damping torque, and damping-like spin-orbit torque (DLT) in terms of *i*-th magnetization $\mathbf{m}_{i}$. Here, $\gamma$ is the gyromagnetic ratio, $\alpha$ is the damping parameter, and $c_{j}$ is the DLT coefficient. The effective field $\boldsymbol{H}_{eff,i}\left( \boldsymbol{=-}H_{\mathrm{ex}}\sum{\hat{\mathbf{m}}}_{j}\boldsymbol{+}H_{k}m_{i,z}{\hat{\boldsymbol{e}}}_{K}+H_{D}\sum\left( -1 \right)^{j+1}{\hat{\mathbf{m}}}_{j}\boldsymbol{\times}\hat{\mathbf{y}} \right)$ consists of antiferromagnetic exchange interactions, easy-axis anisotropy, and an interfacial Dzyaloshinskii-Moriya interaction (iDMI) with their magnitudes denoted by $H_{\mathrm{ex}}, H_{k}$, and $H_{D}$. Here, ${\hat{\mathbf{m}}}_{j}$ is the nearest-neighbor magnetization vector, and ${\hat{\boldsymbol{e}}}_{K}$ aligns with an easy-axis direction of the NiO antiferromagnetic system ([112] family). The DLT coefficient $c_{j}$ is defined as $\hbar\theta_{\mathrm{SH}}J_{c}/2eM_{s}t_{\mathrm{AFM}}$, where $\theta_{\mathrm{SH}}$ is the spin Hall angle, $J_{c}$ is the charge current density in the NM layer, $M_{s}$ is the saturation magnetization of AFM, and $t_{\mathrm{AFM}}$, is the AFM thickness. Spin polarization of DLT is along $\hat{\mathbf{y}}$, and charge current density is along $\hat{\boldsymbol{x}}$. Figure S18 schematically illustrates the spin-current generation and spin pumping mechanism in the Ta/NiO/CoFeB structure used in our experiments. In this system, the charge current in Ta generates a transverse spin current via the spin Hall effect, which drives the dynamics of the antiferromagnetic moments in NiO and induces spin pumping back into the adjacent layers. Note that the $\hat{\boldsymbol{x}}\boldsymbol{,}\hat{\mathbf{y}}$, and $\hat{\boldsymbol{z}}$ axes correspond to $\left[ \bar{1}10 \right],\left[ 001 \right]$ and $[110]$ directions, and $\varphi$ is azimuthal angle from the $\hat{\boldsymbol{x}}$.

We calculated effective spin Hall angles from NiO spin pumping, $\theta_{\mathrm{sp},i}$, defined as $2eJ_{sp,i}/\hbar J_{c}$ where *i* = *x, y*, and *z*, and $\mathbf{J}_{sp,i}=g_{r}^{\uparrow\downarrow}\hbar/8\pi\sum\left( \mathbf{m}_{1}\times\partial_{t}\mathbf{m}_{1}+\mathbf{m}_{2}\times\partial_{t}\mathbf{m}_{2} \right)_{i}$.^[24,31]^ Here $g_{r}^{\uparrow\downarrow}$ is the spin mixing conductance. $\theta_{\mathrm{sp},i}$ demonstrates the conversion efficiency of spin pumping via $\mathbf{J}_{sp,i}=\frac{\hbar}{2e}\theta_{\mathrm{sp},i}\mathbf{J}_{c}$. The simulation parameters are $\gamma=1.76\times{10}^{11} T^{-1}s^{-1}, \alpha=0.001, H_{\mathrm{ex}}=58.7 T, H_{k}=0.64 T,\theta_{\mathrm{SH}}=0.2, J=3\times{10}^{8}A/cm^{2},t_{\mathrm{AFM}}=2 \mathrm{nm}, M_{s}=341 \mathrm{emu}/cc, g_{r}^{\uparrow\downarrow}={10}^{14} \mathrm{cm}^{-2}$ and the system dimensions are $40\times0.4\times2 nm^{3}$ with a unit cell $0.4\times0.4\times2 nm^{3}.$ Here, $H_{\mathrm{ex}}$ represents the antiferromagnetic exchange interaction between Ni sublattices in NiO, which is isotropic and therefore independent of the orientation of the Néel vector, and *M*_s_ corresponds to the magnetization of an individual Ni sublattice. The sublattices are compensated, resulting in zero net magnetization. We assume a large iDMI at NM/antiferromagnet interface, where the effective field amounts to 10% of the effective field of exchange interaction. Chiral texture of NiO induced by iDMI is needed to reverse the polarity of the $\theta_{\mathrm{sp},z}$ upon reversal of the charge current direction (i.e., $\theta_{\mathrm{sp},z}\left( \varphi\right)\approx-\theta_{\mathrm{sp},z}\left( \varphi+180^{\circ} \right)$).

Figures S19a-c present the time average effective Hall angles, $\left\langle\theta_{\mathrm{sp},i} \right\rangle$, for the *x*-, *y*- and *z*- components of the spin-pumping spin currents, respectively. Note that spin current of *y*-spin polarization includes the spin Hall effect contribution in the Ta (we assume a spin Hall angle of 0.2 for Ta) as shown Figure S19b. The overall *φ*-dependence in Figure S19 agrees with the angular dependence of the damping-like effective fields extracted from the harmonic Hall measurements, as presented in Figures 4d-f.

Figure S20 shows the angular dependence of the *z*-component effective spin Hall angle, $\theta_{sp,z}$, for four possible easy axes of the NiO (110) plane: $\left[ 12\bar{1} \right], \left[ 21\bar{1} \right], \left[ 211 \right]$ and $\left[ 121 \right]$. This demonstrates that each axis exhibits a cosine-like function with a 90°-like phase shift relative to each other. This behavior is consistent with the angular dependencies of the field-free SOT switching results in Figure S10e. When the current direction becomes collinear with any of these easy axes, $\theta_{\mathrm{sp},z}$ vanishes at specific angles (e.g., 55°, 125°, 235°, 305°), which corresponds to a mirror-symmetric state of the system.

Figure S18. Schematic illustration of the spin current generation mechanism in the NiO/Ta/CoFeB trilayer based on antiferromagnetic (AFM) spin dynamics. Here, J_c_ denotes the injected charge current, J_s_ represents the spin current generated in Ta, which exerts a torque on the AFM moments in the NiO layer, and J_sp_ is the spin current generated by the AFM dynamics in NiO. M_1_ and M_2_ denote the two AFM sublattices of NiO.

**Figure S19.** a-c) $\left\langle\theta_{\mathrm{sp},i} \right\rangle$ versus *φ* for the NiO with the [211] easy axis for (a) *x*- , (b) *y*- (c) *z*-components.

Figure S20. $\left\langle\boldsymbol{\theta}_{\mathbf{sp}\boldsymbol{,z}} \right\rangle$ versus *φ* for the NiOwith $\left[ \boldsymbol{12}\bar{\boldsymbol{1}} \right]$, $\left[ \boldsymbol{21}\bar{\boldsymbol{1}} \right]\boldsymbol{,}\left[ \boldsymbol{211} \right]\boldsymbol{,}$ and $\left[ \boldsymbol{121} \right]$ easy axes.

**Supporting Information 10. NiO Thickness dependence of antiferromagnetic ordering**

To examine the effect of NiO thickness (𝑡_NiO_) on antiferromagnetic ordering of the NiO layer, we measured the temperature dependence of the coercive field (𝐻_c_) and exchange bias field (𝐻_ex_) in NiO (𝑡_NiO_)/CoFeB bilayers with 𝑡_NiO_ = 0, 5, 15, and 30 nm. Figures S21a and S21b show the temperature dependence of 𝐻_c_ and 𝐻_ex_, respectively. As 𝑡_NiO_ increases, both 𝐻_c_ and 𝐻_ex_ increase, indicating that antiferromagnetic ordering is enhanced in thicker NiO layers.

Figure S21. a, b) Temperature dependence of coercivity (𝐻_c_) and exchange bias field (𝐻_ex_) for NiO (𝑡_NiO_)/CoFeB bilayers: (a) 𝐻_c_ and (b) 𝐻_ex_.

References

[S1]	F. L. A. Machado, P. R. T. Ribeiro, J. Holanda, R. L. Rodríguez-Suárez, A. Azevedo, S. M. Rezende. “Spin-Flop Transition in the Easy-Plane Antiferromagnet Nickel Oxide,” *Physical Review B* 95, no. 10, (2017): 104418. <https://doi.org/10.1103/PhysRevB.95.104418>.
